# In vitro antimicrobial effect of essential tea tree oil(*Melaleuca alternifolia*), thymol, and carvacrol on microorganisms isolated from cases of bovine clinical mastitis

**DOI:** 10.1080/23144599.2022.2123082

**Published:** 2022-09-29

**Authors:** Lysett Corona-Gómez, Laura Hernández-Andrade, Susana Mendoza-Elvira, Feliciano Milián Suazo, Daniel Israel Ricardo-González, David Quintanar-Guerrero

**Affiliations:** a Laboratorio de Posgrado en Tecnología Farmacéutica, FES-Cuautitlán, Universidad Nacional Autónoma de México, Cuautitlán Izcalli, México; b Departamento de Bacteriología del Centro Nacional de Investigación Disciplinaria en Salud Animal e Inocuidad del Instituto Nacional de Investigaciones Forestales, Agrícolas y Pecuarias, Cuajimalpa de Morelos, Cuautitlán Izcalli, México; c Laboratorio de Microbiología y Virología de las Enfermedades Respiratorias del Cerdo, FES-Cuautitlán, Universidad Nacional Autónoma de México, Cuautitlán Izcalli; d Facultad de Ciencias Naturales, Universidad Autónoma de Querétaro. Querétaro, México; e Departamento de Rumiantes, Facultad de Medicina Veterinaria y Zootecnia, Universidad Nacional Autónoma de México, Coyoacán, México

**Keywords:** Bovine mastitis, essential oils, *Melaleuca alternifolia*, thymol, carvacrol

## Abstract

Both Gram-negative and Gram-positive bacteria have recently developed antibiotic resistance to treatments for bovine mastitis, creating a serious concern for public and animal health. The objective of this study was to analyse *in vitro* microbicidal activity of tea tree oil, thymol and carvacrol (composed of oregano and thyme essential oils) on bacteria isolated from clinical mastitis. Field isolates and ATCC strains of the *Staphylococcus* spp, *Streptococcus* spp, *Escherichia coli, Klebsiella pneumoniae, and Candida albicans* genera were analysed. The agar diffusion technique was used to test bactericidal susceptibility and plate microdilution was utilized to determine the minimum inhibitory, bactericidal, and fractional inhibitory concentrations. Thymol alone and the combinations of thymol-carvacrol and thymol-TTO obtained the highest inhibition diameters for Gram-negative bacteria, while for Gram-positive bacteria and *C. albicans*, thymol and the combination thymol-carvacrol obtained the highest indices. TTO, thymol, and carvacrol had MIC values of 1.56–25 mg/ml, 0.05–0.4 mg/ml, and 0.02–0.2 mg/ml, respectively. CMB results for the Gram-negative and gram-positive groups were 0.39–0.78 mg/ml, and for *C. albicans*, 0.78–1.56 mg/ml. Results for the fractional inhibitory concentrations show that the TTO+thymol and thymol+carvacrol combinations had additive activity against groups of Gram-negative bacteria and *C. albicans*. These natural components, evaluated individually and in combinations, have an effectiveness above 70%.

## 1. Introduction

Bacteria-related bovine mastitis are a main cause of antimicrobial usage in dairy cattle. In addition to causing economic losses due to wasted milk, mastitis can contribute to the development of bacterial resistance to antibiotics [1–3, 7]. In recent years, some of the aetiological agents that cause mastitis have shown partial or total resistance to various antimicrobial treatments [4–6, 8], so the World Organization for Animal Health supports research on alternative treatments to antibiotics [9]. Defined as inflammation of the mammary gland, mastitis has a severe economic impact on dairy cattle production units. It occurs through interaction among the causal agent, the individual animal, and the environment in which it lives [10]. The most important contagious pathogens are *Staphylococcus aureus, Streptococcus agalactiae, Corynebacterium* spp. and *Mycoplasma* spp [11].

Most intramammary infections caused by these bacteria are subclinical, but they can evolve into chronic infections diagnosed as clinical mastitis [11]. Environmental pathogens include *Streptococcus uberis* and *Escherichia coli* [11]. While the costs of subclinical mastitis are difficult to quantify, most experts agree that it impacts the average dairy farmer more than clinical mastitis, largely due to irreversible damage to the udder tissue. Mastitis is also the leading cause of antibiotic use in dairy production [12], so there is an urgent need for research on alternative antimicrobial agents. In this regard, essential oils (EO) and some of their components have been proposed as viable options that are gaining interest in various fields of human and veterinary medicine [13].

Essential oils are characterized by high concentrations (20–70%) of two or three main components that may have various applications, including as antimicrobial agents [14]. The bactericidal effects of essential oils have been evaluated for use as options either alone or combined with drugs to obtain beneficial synergies [15,16].

The bactericidal effect of tea tree oil (TTO) is attributed primarily to terpinen-4-ol, its principal component [17]. Carvacrol and thymol are terpenoid components found in high proportions in the essential oils of oregano and thyme. They are recognized as safe (GRAS) by the Food and Drug Administration (FDA) [18]. Several studies have suggested that the antibacterial mechanism of thymol and carvacrol could be a consequence of the disturbance of the lipid fraction of the bacterial plasma membrane that alters permeability and allows intracellular material to escape [15,18,19]. Recent observations show that thymol and carvacrol not only inhibit the biofilm production of some gram-positive bacteria (e.g. *Streptococcus mutans* [20]) but also have bactericidal activity against bacteria that are resistant to some antibiotics [21]. Currently, the use of essential oils and their components is being investigated for the development of strategies to control bovine mastitis [13], both as adjuvants for vaccines [22,23] and as antimicrobials [2,6,24–27]; in some studies, they are evaluated against multidrug-resistant strains isolated from bovine mastitis [28,29], their potential antioxidant and antibiofilm effects have also been studied [2,30,31]. The aim of this research is to analyse the *in vitro* microbicidal activity of tea tree oil (TTO, *Melaleuca alternifolia*), thymol, and carvacrol against field isolates and ATCC strains of *Staphylococcus* spp, *Streptococcus* spp, *Escherichia coli, Klebsiella pneumoniae and Candida albicans* isolated from cases of clinical mastitis.

## 2. Methodology

### 2.1. Essential oils and extracts

Tea tree essential oil (Newsystec, Lot1922151) and thymol and carvacrol extracts (98% Sigma Aldrich) were used. Tea tree oil, whose AE is extracted comes from steam distillation of the species “*Melaleuca alternifolia*” native to Australia, by steam extraction from the leaves and bark.

Chromatography in conjunction with gas chromatography-mass spectrometry (CG-EM) was used to determine the chemical composition of the EO of tea tree oil. The main ingredients identified are (Newsystec, batch 2023313): Terpinen-4-ol (39.1%), a-Pinene (2.6%), Sabinene (0.3%), α-Terpinene (9.2%), Limonene (1%), p-Cymene (3.2%), 1,8 Cineole (2.9%), γ-Terpinene (21.1%), a-Terpinolene (3.4%), α-Terpineol (2.9%), Aromadendrene (1.4%), δ-Cadinene (1.1%), Globulol (0.2%), Viridiflorol (0.2%). The qualitative determination is transparent liquid, colourless light yellow; density at 20°C 898 g/mol, refractive index at 20°C 1.4764.

### 2.2. Strains

The following bacterial strains were used: *S. aureus* ATCC BAA976, lot 365–90-5, MediMark®Europe, *S. aureus*, field strain isolated from case of clinical mastitis, *S. aureus* Cowan strain, *Staphylococcus epidermidis*, field strain isolated from a case of clinical mastitis, *Streptococcus pyogenes* ATCC 19615, MediMark®Europe, France, *Streptococcus uberis*, field strain isolated from a case of clinical mastitis, *Streptococcus dysgalactiae*, field strain isolated from a case of clinical mastitis, *E. coli ATCC 8739* MediMark®Europe, France, *E. coli*, field strain isolated from a clinical case of mastitis, *K. pneumoniae ATCC 700603, K. pneumoniae*, field strain isolated from a case of clinical mastitis, *C. albicans* ATCC 14053, and *C. albicans*, field strain isolated from a case of clinical mastitis.

### 2.3. Evaluation of the bactericidal sensitivity of the essential oils

Bactericidal sensitivity was determined by the agar diffusion technique following the method described by Kirby-Bauer [32]. Filter paper discs of approximately 6 mm were impregnated individually with 20 µl of TTO, thymol, and carvacrol and combinations of these at a 1:1 ratio and stored in refrigeration until use. An impregnated disc was placed on the surface of Müller-Hinton agar previously seeded with the bacterial dilution of 10^8^ CFU/ml, equivalent to 0.5 of the McFarland standard and 0.1 of absorbance at a wavelength of 600 nm. Discs were incubated for 24 h at 35°C in a bacteriological oven. Ciprofloxacin and ceftiuofur, respectively, were used as positive controls for gram-negative and gram-positive bacteria, while 2% dimethyl sulphoxide (DMSO) was the negative control for both groups of bacteria. For *C. albicans*, itraconazole (30 µg) and 2% DMSO were used as the positive and negative controls. Measurement of the inhibition halos was performed with vernier. All assays were performed in triplicate. The percentage of inhibition was obtained from the measurements using the formula described by Cruz-Carrillo et al. (2010) [33,34].

$$\;\;\%\;of\;inhibition=\frac{inhibition\;halo\;diameter}{positive\;control\;halo\;diameter}*100$$

Based on the results of this equation, antibacterial activity can be classified as high, when the percentage of relative growth inhibition is >70%, intermediate, between 50 and 70%, and low when it is <50%[33].

### 2.4. Determining minimum inhibitory and bactericidal concentrations

A stock solution of TTO was prepared at 800 mg/ml, based on the density of the oil and diluted with dimethyl sulphoxide (DMSO). Thymol and carvacrol were diluted in ethanol to obtain a concentration of 50 mg/ml as a stock solution. The microplate dilution reference method was used to determine the minimum inhibitory concentrations (MIC) following the Clinical and Laboratory Standards Institute guidelines [35]. The strains were seeded 12 h before testing, standardized with the 0.5 McFarland tube, corroborated with an optical density reader, and adjusted to an absorbance of 0.1 at a wavelength of 600 nm. For testing, 100 µL of the inoculum were sown on brain-heart infusion agar (BHI) and incubated at 37°C for 24 h, when the colonies were counted. Using a 96-well microplate, double dilutions of TTO, thymol, and carvacrol were made with brain-heart broth. Then, 20 µL of the inocula of the *Staphylococcus* spp, *E. coli, Klebsiella* strains, and *C. albicans* were placed, but 30 µL were used for *Streptococcus* spp. Evaluation was based on growth inhibition on the plate. Assays were performed in triplicate. The minimum inhibitory concentration was determined using the well in which no button was observed. A growth control of the inocula was placed as a positive control. DMSO was used as a negative control.

### 2.5. Determining the minimum bactericidal concentration

This was determined by the colony count method of the dilutions in which no buttons were observed. The inoculum was diluted to a concentration of 1:1000 in saline solution, then 100 µL of the dilution were placed in BHI agar, incubated at 37°C for 24 h. At that point, the colonies were counted.

### 2.6. Determining the fractional inhibitory concentration index

Inhibitory interaction studies were performed using the chequerboard technique [36–38]. **The antibiotic agents were added individually and in combinations. The fractional inhibitory concentration index (FIC) was calculated using the following equation:

$$FICI=\overset{n}{\underset{i=1}{\sum }}\frac{MIC\;concentrations\;of\;the\;drugs\;in\;combination}{MIC\;concentrations\;of\;the\;drugs\;alone}$$

When interpreting the results, a synergistic condition was considered when FICI was ≤0.5, an additive condition when it was ≥0.5 and ≤1, an indifferent condition when it was >1 and ≤4, and an antagonistic condition when it was >4.

### 2.7. Statistical analysis

Analysis of variance (ANOVA) tests and a post hoc Tukey test were used for data analysis.

## 3. Results

### 3.1. Evaluation of the bactericidal sensitivity of the essential oils by the plate diffusion technique

**Figure 1.**
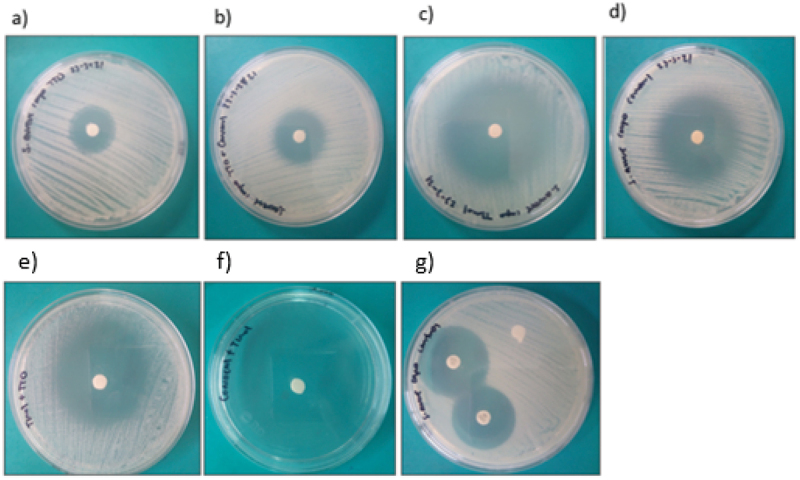
Inhibition halos of the different natural extracts against gram-positive bacteria of the genus *Staphylococcus* spp.

**Figure 2.**
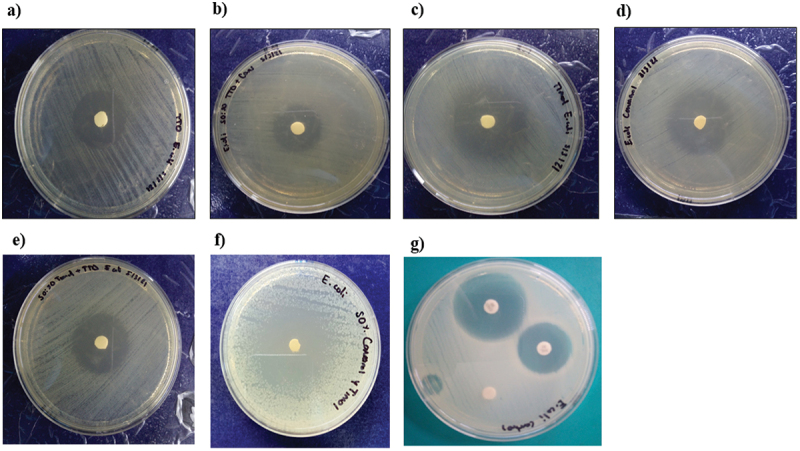
Inhibition halos of the different natural extracts against gram-negative bacteria (*E. coli).*

**Figure 3.**
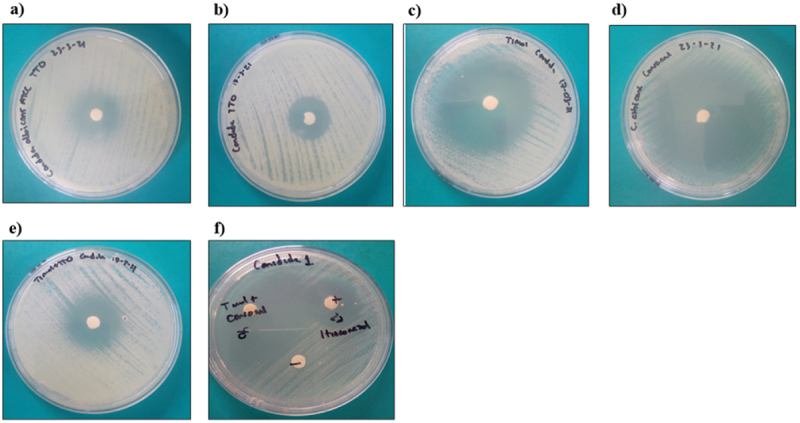
Inhibition halos of the different natural extracts against *Candida albicans.*

Thymol and the combination thymol+carvacrol had the largest inhibition halo diameters (mm) for the gram-positive bacteria 42.2 ± 5.5 and 30.8 ± 8.7 (Figure 1). Thymol and the combinations thymol+carvacrol and TTO+thymol had larger inhibition halo diameters (mm) against the gram-negative bacteria at 31.2 ± 0.7, 23.4 ± 1.9, and 29.11 ± 1.3, respectively (Figure 2). Thymol and the combination thymol+carvacrol had the largest inhibition halo diameters (mm) for the yeasts 43.8 ± 0.2 and 44.5 ± 1.6, respectively (Figure 3). These results indicate that TTO, thymol, carvacrol, and the combinations TTO+thymol, TTO+carvacrol, and thymol+carvacrol had bacterial inhibition percentages above 70% against all the strains evaluated compared to controls.

a) TTO, tea tree oil; b) TTO+carvacrol; c) thymol; d) carvacrol; e) TTO+thymol; f) thymol+carvacrol; g) positive controls (ciprofloxacin, ceftiofur) and the negative control.

a) TTO; tea tree oil; b) TTO+carvacrol; c) thymol; d) carvacrol; e) TTO+thymol; f) thymol+carvacrol; g) positive controls (ciprofloxacin, ceftiofur) and the negative control.

a) TTO; tea tree oil; b) TTO+carvacrol; c) thymol; d) carvacrol; e) TTO+thymol; f) thymol+carvacrol; positive control (itraconazole (30 mg)) and the negative control.

### 3.2. Minimum inhibitory and bactericidal concentrations

Results for the MIC of the TTO for the gram-negative bacteria were 0.78–3.13 mg/ml, for thymol, 0.1–0.2 mg/ml, and for carvacrol, 0.02–0.2 mg/ml. For the gram-positive bacteria, values were 3.13–25 mg/ml of TTO, 0.1–0.2 mg/ml of thymol, and 0.02–0.4 mg/ml of carvacrol. For *C. albicans*, results were 6.25 mg/ml of TTO, 0.05–0.4 mg/ml of thymol, and 0.1–0.2 mg/ml of carvacrol.

Results for the CMB of the TTO for the gram-negative bacteria were 6.25–12.50 mg/ml, for thymol 0.4 mg/ml, and for carvacrol, 0.1–0.7 mg/ml. For the gram-positive bacteria, the figures were 6.25–52 mg of TTO, 0.2–0.4 mg/ml of thymol, and 0.2–0.78 mg/ml of carvacrol. For *C. albicans*, results were 6.25–12.50 mg/ml of TTO, 0.4–1.6 mg/ml of thymol, and 0.4–0.8 mg/ml of carvacrol.

### 3.3. Determining the fractional inhibitory concentration index

The TTO-thymol combination produced additive activity in the group of gram-negative bacteria and *C. albicans*. The TTO-carvacrol combination generated a result of indifference with all three bacterial groups. The thymol-carvacrol combination had additive activity for the group of gram-negative bacteria and *C. albicans* (Table 1).

**Table 1.** Indicates the determination of the fractional concentration (IFIC) of the combinations of natural active ingredients by group of bacteria and interpretation.

	Combinations						
	TTO+thymol			TTO+carvacrol		Thymol+carvacrol	
Strain groups	Mean IFIC		Interpretation	Mean IFIC	Interpretation	Mean IFIC	interpretation
Gram-negative	A	0.98^B^	Additive	1.05^B^	Indifferent	0.8^B^	Additive
Gram-positive	B	1.3	Indifferent	1.96	Indifferent	1.34	Indifferent
*C. albicans*	C	1	Additive	1.5	Indifferent	0.9	Additive

Different literals in each column indicate a significant difference (p < 0.05).

## 4. Discussion

The use of EOs is aimed to counteract the effects of multi-drug resistance to antibiotics, as well as to reduce or otherwise penetrate biofilm which achieve a bactericidal effect and thus enable more effective antimicrobial treatment. Currently, essential oils have become very important, especially in veterinary medicine, where alternative treatments for infectious diseases are sought. However, there are few *in vivo* studies in dairy cows. Abboud et al. (2015), studied the use of two EOs, *Thymus vulgaris*, and *Lavandula angustifolia*, by administering a 10% solution of the oils intramammary and found a drastic decrease in the number of bacterial colonies in the different milk samples after four consecutive treatments [1,39]. Research approaches the use of EOs are aimed to counteract the effects of multi-resistance to antibiotics, so its effects have been studied *in vitro* in combination with antibiotics or other essential oils. However, the combination of essential oil (TTO) and individual components of other oils, e.g. thymol or carvacrol, has hardly been studied [4]. There are few studies investigating the antimicrobial activity of tea tree essential oil and its behaviour in combination with thymol and carvacrol, components with bactericidal activity of oregano and thyme essential oils. Kang Zhan et al.(2020) [40,41], **investigated the effect of TTO on bovine mammary gland epithelial cells and proinflammatory cells, finding that a concentration of 0.025% and 0.05% of TTO promoted polymorphonuclear proliferation and epithelial cells viability against *S. aureus* infection. On the other hand, Zhi Chen et al. (2020) showed that TTO can promote autolysis of *E. coli* and exert a remarkable inhibitory effect on LPS-induced inflammation, and the proportion of normal living mammary epithelial cells stimulated by LPS increased after treatment with TTO at a concentration (<50 μg/ml LPS). Similarly, the proportion of early apoptosis, late apoptosis, and dead cells decreased as TTO attenuated LPS-induced TNF-α and IL-6 expression [42]. Earlier studies have shown that TTO acts as a membrane permeabilizer and causes a loss of chemiosmotic control in gram-positive and gram-negative bacteria [43], and damages the membrane of *C. albicans* [44]. The bactericidal effect of TTO is due primarily to the terpinen-4-ol component that exists in a high proportion in this oil. TTO has been studied in goats as an active ingredient in teat disinfection formulations applied to prevent mastitis, obtaining an efficacy equivalent to that of common commercial disinfectants [45]. *S. aureus* causes especially severe economic losses but eliminating it with antibiotics is especially challenging due to its ability to invade mammary gland cells, quickly develop resistance mechanisms to numerous antibiotics, and generate biofilm formation. Some studies have shown that terpinen-4-ol can inhibit biofilm formation in *S. aureus* [46]. Thymol and carvacrol are isomers with hydroxyl groups at different positions. They are components of essential oils of thyme and oregano that have been evaluated extensively and shown evidence of interaction with bacterial cell membranes that affects their permeability due to the loss of membrane potential caused by the leakage of potassium ions, ATP, and carbohydrates [47]. The MIC ranges of thymol and carvacrol obtained in our study agree with those reported in earlier works that evaluated Gram-positive and Gram-negative bacteria [10,35,48,49].

Table 1 indicates the determination of the fractional concentration, an additive activity was observed for the TTO +thymol and thymol+carvacrol combinations for the group of coliform bacteria and *C. albicans*, but not for the TTO+carvacrol combination, which had an activity of indifference, as did all three combinations for the group of Gram-positive bacteria. Differences in susceptibility between *E. coli* and *S. aureus*, and to some extent *C. albicans*, can be explained by differences in the extent of cell membrane damage which induced by monoterpenes. There is indifferent activity which may be due to the hydrocarbon components of TTO are the main active ingredients playing an antagonistic role. This was supported by Cox et al. [43] who found that terpinene-4-ol alone was significantly more active than a tea tree oil dispersion containing an equivalent amount of terpinene-4-ol. They observed that *S. aureus* was sensitive to TTO in MIC assays at levels comparable to *E. coli* and *C. albicans*; however, time-kill studies revealed that *S, aureus* dies more slowly [43]. Therefore, it appears that monoterpene hydrocarbons offer the most significant antagonistic effects against microorganisms that are not rapidly killed by TTO. Little is known today about the factors that govern synergy and antagonism among the components of essential oils. Four theoretical mechanisms exist to describe the antimicrobial interactions that produce synergy: (i) sequential inhibition of several steps in a specific biochemical pathway; (ii) inhibition of the degradation enzymes of microorganisms; (iii) interaction of several antimicrobials with the wall of the bacterial cell; and (iv) interaction with the cell wall or membrane that increases absorption of antimicrobials [47]. Of the over 100 components of TTO, only perhaps 10 have been identified as primary active substances.

One advantage of essential oils (EO) is their apparently low induction of bacterial resistance, possibly because they do not attack one specific target and may have multiple modes of antibacterial action. The presence of several components with antibacterial activity can hinder the development of resistance as a mechanism of pathogenicity in bacteria [15,50]. More research is needed on the interactions of the active components of numerous essential oils and their possible synergies against bacteria and fungi.

## 5. Conclusions

The *in vitro* bactericidal activity of *Melaleuca alternifolia* tea tree oil (TTO), thymol, and carvacrol against field isolates and ATCC strains of *Staphylococcus* spp, *Streptococcus* spp, *Escherichia coli, Klebsiella pneumoniae*, and *Candida albicans* isolated from clinical mastitis were evaluated. Of the natural oils tested, thymol had the largest inhibition halo diameter for most of these strains. The combinations of thymol+carvacrol and TTO+thymol showed additive activity with the group of gram-negative bacteria and *C. albicans. In vitro* testing of natural active ingredients has revealed inhibition rates above 70% compared to positive controls. This indicates that the combinations of thymol+carvacrol and TTO+thymol can be used to develop formulations as alternatives to conventional antimicrobial therapy for bovine mastitis, or to improve the efficacy of existing treatments. TTO, thymol, and carvacrol should also be evaluated for other pathologies caused by bacterial or fungal infections that affect cattle. In fact, they are being studied in multidisciplinary research laboratories for various applications, including as preservatives for food and cosmetics. Finally, TTO has attracted attention because of its anti-inflammatory, antiseptic, disinfectant, antiviral, and anticancer properties.
